# Obesity management: sex-specific considerations

**DOI:** 10.1007/s00404-023-07367-0

**Published:** 2024-02-08

**Authors:** Tobias Kantowski, Clarissa Schulze zur Wiesch, Jens Aberle, Anne Lautenbach

**Affiliations:** The University Obesity Center, University Medical Center Hamburg-Eppendorf, Martinistr 52, 20246 Hamburg, Germany

**Keywords:** Obesity, Conservative therapy, Pharmacotherapy, Bariatric surgery, Sex-specific differences

## Abstract

Obesity is a global health issue that has grown to epidemic proportions. According to World Health Organisation (WHO), overweight and obesity are responsible for more than 1.2 million deaths in Europe each year, representing > 13% of the region's total mortality. Highly processed, calorie-dense foods and reduced physical activity are considered as primary drivers of obesity, but genetic predisposition also plays a significant role. Notably, obesity is more prevalent in women than in men in most countries, and several obesity-related comorbidities exhibit sex-specific pathways. Treatment indication depends on BMI (body mass index), as well as existing comorbidities and risk factors. To reduce obesity-associated comorbidities, a permanent reduction in body weight of (at least) 5–10% is recommended. Treatment guidelines suggest an escalating stepwise approach including lifestyle intervention, pharmacotherapy, and bariatric-metabolic surgery. As cumulative evidence suggests differences in weight loss outcomes, there is growing interest in sex-specific considerations in obesity management. However, most trials do not report weight loss or changes in body composition separately for women and men. Here, we discuss state-of-the-art obesity management and focus on current data about the impact of sex on weight loss outcomes.

## Introduction

Obesity is a complex global health issue due to a combination of causes and individual factors such as sex, sociocultural, environmental, genetic and physiological factors. The increasing availability of highly processed, high-calorie food combined with reduced physical activity is considered main drivers for the development of obesity. In addition, there are predisposing genetic factors that lead to impaired energy homeostasis. Since overweight and obesity per se are associated with an increased risk of coronary heart disease, cerebrovascular disease, and heart failure [1], obesity leads to a significant reduction in lifespan in men and women [2].

Obesity is more prevalent in women than men in most countries and several obesity-related comorbidities demonstrate sex-specific pathways [3]. Based on neuroimaging studies, women show a greater neural response to highly palatable, energy-dense foods favoring overweight and obesity [4]. Sex-specific obesity phenotypes identified by validated biological and behavioral testing of key components of energy balance suggest a phenotype-guided obesity-pharmacotherapy in men and women given physiological and biological differences between sexes [5].

Based on the current evidence, here we focus on state-of-the-art obesity management including sex-specific weight loss outcomes in response to lifestyle intervention, pharmacotherapy and bariatric-metabolic surgery.

## Treatment indication

**Fig. 1:**
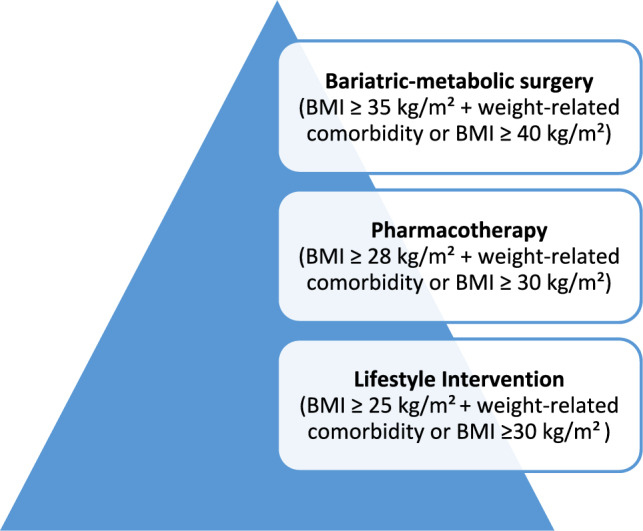
Interventions in obesity management depending on BMI and comorbidities

Whether treatment for overweight and obesity is indicated depends on BMI (body mass index), as well as existing comorbidities and risk factors **(**Fig. 1**)**. Treatment is indicated for patients with a BMI ≥ 30 kg/m^2^ or ≥ 25 kg/m^2^ with weight-related comorbidity, visceral obesity, high levels of psychosocial distress or diseases that are aggravated by high body weight [6].

The sustainability of weight reduction is one of the major challenges in obesity management. The upcoming S3 guideline of the German Obesity Society will devote a separate chapter to “weight loss maintenance”. Therefore, obesity management is not about temporary diets, but about continuous lifestyle modification. Patient education about the chronicity of the disease is an important element of effective long-term management.

## Lifestyle modification

Lifestyle modification consisting of dietary, exercise and behavioral therapy is a fundamental component of obesity management and should always be applied early and consistently. The repeated and coordinated implementation of nutritional, exercise and behavioral therapy interventions in an interdisciplinary team is crucial.

Current guidelines stress the importance of personalized weight management. Therefore, individual assessment of eating habits, preferences and metabolic goals is crucial. Current evidence indicates that macronutrient composition is not decisive for long-term weight loss, but rather a calorie-reduced diet [6]. Energy intake should be reduced in a controlled manner after standardized evaluation of eating behaviors taking into account concomitant diseases. A daily energy deficit of at least 500 kcal should be achieved.

The efficacy of the Mediterranean diet for the prevention of cardiovascular disease has been demonstrated in the PREDIMED study [7]. In the CORDIOPREV study, the largest study to date on the efficacy of the Mediterranean diet in secondary prevention, 1002 patients with coronary heart disease were randomized in a 1:1 ratio to receive either a Mediterranean diet or a low-fat dietary intervention [8]. During the 7-year study period, the Mediterranean diet showed superior efficacy and led to a 26% greater risk reduction in the primary endpoint (composite of major cardiovascular events including myocardial infarctions, necessary revascularizations, ischemic strokes and peripheral arterial disease, and cardiovascular-related deaths).

Different types of intermittent fasting have become a popular strategy to restrict calorie intake. While short-term positive effects on weight loss (< 6 months) and improvements in cardiometabolic risk parameters have been found, intermittent fasting seems not to be superior to continuous caloric restriction in the long-term (12 months) [9]. In fact, intermittent fasting can cause greater lean mass loss due to reduced dose and frequency of protein consumption per meal [10].

The guidelines contain recommendations about the short-term use of meal replacement product-based diets (“formula diets”) for weight control. Total energy intake should be limited to 800–1200 kcal/day for enabling weight loss of approx. 0.5–2.0 kg/week over a period of up to 12 weeks. In adults with type 2 diabetes (T2D), low energy diets with formula meal replacement are considered to be the most effective dietary intervention for weight management and remission of T2D as shown by the DiRECT trial (Diabetes Remission Clinical Trial). In addition to an average reduction in body weight of 10 ± 8.0 kg in 12 months, remission of T2D was achieved in 46% of subjects in the intervention group [11]. Moreover, formula diets are considered to be one of the most effective strategies for weight maintenance following successful weight loss in T2D [12].

## Physical activity

Implementing exercise into everyday life is of importance for the prevention of obesity, but even more important for weight maintenance after successful weight reduction.

Duration and type of physical activity must be individually adjusted. However, for effective weight loss, exercise > 150 min/week with an energy expenditure of 1200–1800 kcal/week is required. If the amount of exercise is 225–420 min/week (1800–3360 kcal/week), weight reduction of 5.0–7.5 kg can be achieved [6]. In particular, activity in the aerobic range and also shorter units of physical activity are beneficial in reducing cardiovascular risk. In addition, it is recommended to engage in muscle-strengthening activities on two or more days, which additionally promotes mobility, strength and increase in muscle mass [6].

Regular physical activity, with or without weight loss, can also improve many cardiometabolic risk factors in adults with overweight or obesity, such as hyperglycemia and insulin sensitivity, arterial hypertension and dyslipidemia. In addition, health-related quality of life, mood disorders (e.g., depression, anxiety) and body image can be improved in adults with overweight or obesity [6].

## Behavioral therapy

A structured analysis of factors (stress, emotions, psychiatric pre-existing/co-existing illnesses, etc.) contributing to pathological eating habits is crucial. In addition to behavioral therapy (e.g., learning coping strategies, applying stimulus control techniques, and adhering to a regular meal structure), strategies for improving self-acceptance, positive body image and dealing with weight-related discrimination are taught. Personal contact with the health care team, regular self-observation and the development of problem-solving strategies are considered to be particularly relevant. Engagement of one’s social network leads to significantly greater change across multiple risk behaviors [13]. Moreover, consistent success in behavior-based obesity treatment is highly related to treatment adherence and may include digital approaches [13].

## Sex-specific considerations

There is growing interest in exploring sex differences in weight loss outcomes resulting from lifestyle interventions. However, males are often underrepresented in lifestyle weight loss trials [14].

According to available data, men tend to lose more weight than women with non-pharmacological lifestyle interventions including low-carbohydrate diets [15], low-fat diets [16], very-low-energy diets [17] and exercise interventions [18]. However, according to a systematic review about differences in the effectiveness of weight loss interventions between men and women, analysis of effect sizes found only small differences in weight loss favoring men for both, diet (*g* = 0.489) and diet plus exercise (*g* = 0.240) interventions [19]. The systematic review identified insufficient evidence that sex-specific weight loss strategies are required. It can be assumed that short-term differences in weight loss outcomes may have little impact long-term. Moreover, data available so far suggest that weight loss interventions may not need to be tailored to women’s menopausal status [20].

Successful weight maintenance is more prevalent in women, possibly driven by a more gradual, steady weight loss (1–2 pounds per week) compared to men [21]. Therefore, moderate energy restriction combined with moderate physical activity is recommended for successful long-term weight loss independent from sex [6].

## Pharmacotherapy

According to the guideline of the German Obesity Society (DAG), which is currently being revised, pharmacotherapy is indicated as part of an escalating stepwise approach if a patient has not lost more than 5% of his or her initial weight within 6 months with lifestyle modification or if weight loss has not been achieved successfully in the long term [6]. Anti-obesity medication should only be considered as adjunct pharmacotherapy in combination with lifestyle intervention (nutrition, exercise, and behavioral therapy).

### Approved pharmacotherapies

Currently, there are three European Medicines Agency (EMA)-approved drugs for the long-term management of obesity in Germany: the lipase inhibitor Orlistat (Xenical^®^), the GLP-1 receptor agonists (GLP-1RA) liraglutide 3 mg once daily (Saxenda^®^) and semaglutide 2.4 mg once weekly (Wegovy^®^).

### Orlistat

Until 2015, the lipase inhibitor orlistat (Xenical^®^) was the only approved drug for the long-term management of obesity. In a meta-analysis of 12 studies, a mean weight reduction of 8% vs. 4% with placebo was achieved within 12 months [22]. The use of orlistat is often limited by an unfavorable benefit-risk profile. Gastrointestinal side effects include flatulence and steatorrhoea.

### Liraglutide

The GLP-1 receptor agonist has been available in the European Union since 2015 for the treatment of obesity in a maximum dose of 3.0 mg (in contrast, liraglutide 1.8 mg is used for the treatment of T2D).

For the management of obesity, liraglutide was investigated in the SCALE (Satiety and Clinical Adiposity—Liraglutide Evidence) Obesity and Prediabetes trial [23]. In total, 3731 patients were included and treated with liraglutide 3 mg or placebo for 56 weeks. Weight loss was 6.1% vs. 1.9% with placebo. Gastrointestinal side effects such as nausea and vomiting are common, especially in the dose titration phase. Therefore, a gradual increase with an initial dosage of 0.6 mg for one week is recommended. The dose can be increased in weekly intervals (1.2 mg, 1.8 mg, 2.4 mg, and 3 mg) up to the maximum dosage of 3 mg once daily. In case of poor tolerance, dose titration may be prolonged and the dose decreased to the maximum tolerated dose.

### Semaglutide

The efficacy and safety of semaglutide 2.4 mg once weekly in the treatment of obesity was investigated in the phase 3 study program STEP (Semaglutide Treatment Effect in People with Obesity). Semaglutide is a long-acting agonist at the human GLP-1 receptor and can be administered subcutaneously once weekly due to its higher albumin affinity compared to liraglutide. In the STEP-1 study, weight loss in 1306 patients with a BMI ≥ 30 kg/m^2^ or ≥ 27 kg/m^2^ with at least one weight-related comorbidity (without T2D) treated with semaglutide 2.4 mg once weekly plus accompanying lifestyle intervention after 68 weeks was 14.9% (placebo group 2.4%) [24]. With a mean initial weight of 105 kg, this corresponds to a weight loss of 15.3 kg after just over 1 year. More than one-third of the subjects in the intervention group in the STEP-1 study lost at least 20% of their initial weight.

The head-to-head trial comparing liraglutide 3 mg vs. semaglutide 2.4 mg (STEP 8) also showed a significantly greater mean weight reduction of 15.8% with semaglutide versus 6.4% with liraglutide [25].

Gastrointestinal side effects are most common during dose titration, so that a stepwise dose titration is recommended. The initial dosage is 0.25 mg once weekly. Every 4 weeks the dosage can be increased (0.5 mg, 1 mg, 1.7 mg, and 2.4 mg) up to the recommended dosage of 2.4 mg once weekly.

## Pharmacotherapy—sex-specific considerations

Similar to lifestyle intervention trials, most pharmacological trials do not report weight loss separately for males and females given the fact that the majority of patients in pharmacological weight loss trials are women. However, sex does affect weight loss outcomes. Recent data about sex differences suggest that women tend to lose more weight than men after taking weight loss drugs for 1 year. As part of the analysis, data from STEP-1, SCOUT (Sibutramine Cardiovascular OUTcomes trial) and SCALE were evaluated [26].

In STEP-1, mean change in weight after 68 weeks of semaglutide in men vs. women was − 12.9% vs.− 18.4% (*p* < 0.05). Mean change in weight after 68 weeks of placebo in men vs. women was − 3.5% vs.− 2.1% (*p* < 0.05). In the SCOUT trial, the effect of sibutramine, a norepinephrine, serotonin and dopamine reuptake inhibitor, on cardiovascular outcomes vs. placebo was investigated for 12 months. In 10.744 adults with overweight or obesity with cardiovascular disease and/or T2D, mean change in weight after 12 months of sibutramine in men vs. women was − 4.0% vs. − 5.2% (*p* < 0.0001). Mean change in weight after 12 months of placebo in men vs women was − 1.9% vs. − 2.2% (*p* < 0.005). In the SCALE trial, mean change in weight after 68 weeks of placebo in men vs women was −3.5% vs. −2.1% (*p* = 0.25). Interestingly, at the same exposure levels, women lost more weight than men with liraglutide 3 mg once daily [27]. Moreover, the researchers analyzed data from the STEP-2 trial of semaglutide comparing the standard 1.0 mg dose versus the 2.4 mg dose in participants with T2D and overweight or obesity over 68 weeks, the CONQUER trial of phentermine and topiramate (Qsymia^®^), and the SCALE Diabetes trial of liraglutide; analyses demonstrated concordant sex differences in response to weight loss drugs.

Overall, GLP-1 receptor agonists present a tolerable safety profile. The most common reported adverse effects are gastrointestinal side effects such as nausea and vomiting. Available evidence suggests that side effects occur at a higher rate in women, especially affecting gastrointestinal (GI) adverse events. A post hoc analysis of two randomized, controlled studies in Japanese patients with T2D treated with dulaglutide 0.75 mg or liraglutide 0.9 mg showed, that overall incidence of treatment-emergent adverse events was higher in females (86.5% of women vs. 61.4% of men for dulaglutide and 83.3% vs. 65.5% for liraglutide, respectively). Gastrointestinal adverse events also occurred more frequently among women in both treatment groups [28]. In addition, an observational, retrospective disproportionality analysis based on the food and drug administration (FDA) pharmacovigilance database reported semaglutide-associated gastrointestinal AEs seemed to predominantly affect females (57.68%). Importantly, there was no statistically significant difference in serious adverse events between women and men [29].

The different response to GLP-1 receptor agonists may be partially explained by increased drug exposure due to the lower average body weight in female study participants, as body weight was shown to have significant effect on pharmacokinetics on GLP-1 receptor agonists [30]. However, a pharmacokinetic study that used samples from the SCALE trial showed a 32% higher (90% CI 28–35) exposure in females than males with the same body weight identifying sex as one of the key covariates for liraglutide exposure besides body weight [31]. An exposure–response analysis that used data from the SUSTAIN (Semaglutide Unabated Sustainability in Treatment of Type 2 Diabetes) program found, that nausea and vomiting were more frequent in women at similar levels of exposure [32]. These findings possibly suggest that there are mechanisms other than drug exposure that cause sex-specific treatment response to GLP-1 receptor agonists.

## Future pharmacological interventions

### GLP-1-based polyagonists

Enhancing incretin action is an important approach to successfully treating T2D and controlling overweight and obesity. Therefore, the development of GLP-1-based polyagonists (bi- and triagonists) that activate different incretin receptors in one molecule to improve efficacy while maintaining an adequate tolerability and safety profile, is a milestone in T2D and obesity management and treatment.

### Tirzepatide

The effectiveness of the GLP-1/GIP co-agonist tirzepatide for obesity management is being investigated in the SURMOUNT study program [33]. In the phase 3 SURMOUNT-1 trial, treatment with Tirzepatide once weekly resulted in a mean weight reduction of up to 20.9% over a period of 72 weeks approaching weight loss induced by sleeve gastrectomy [34]. The side effect profile is similar to that of GLP-1 receptor agonists, whereas the GIP agonism appears to attenuate nausea induced by the GLP-1 receptor agonism. Approval for the treatment of obesity is not expected before 2024 in Germany.

## Bariatric-metabolic surgery

### Indication

There is convincing evidence for efficacy and safety of bariatric-metabolic surgery over a period of about 20 years [35, 36].

According to the S3 guideline “Surgery for obesity and metabolic diseases”, bariatric surgery is recommended for patients with a BMI of ≥ 40 kg/m^2^ (without the presence of obesity-associated comorbidities) or ≥ 35 kg/m^2^ in the presence of obesity-associated comorbidities if lifestyle intervention has failed [37]. Under certain circumstances, a surgical procedure may be chosen primarily without a prior attempt of lifestyle modification (“primary indication”). These include

- Patients with a BMI of ≥ 50 kg/m^2^
- Patients for whom a conservative therapy attempt is not considered promising by the multidisciplinary team
- Patients with particularly severe concomitant and secondary diseases that do not permit delay of surgical intervention (e.g., patients prior to organ transplantation).

- Contraindications to bariatric-metabolic surgery include:
  - Unstable psychopathological conditions, untreated bulimia nervosa, active substance dependence
  - Underlying consumptive diseases, malignant neoplasms, untreated endocrine causes, chronic diseases worsened by postoperative catabolic metabolism
  - Pre-existing or imminently planned pregnancy

Metabolic surgery, which is performed in order to improve glycemic control in patients with pre-existing T2D, should also be primarily recommended for patients with BMI ≥ 40 kg/m^2^ (“primary indication”).

Perioperative mortality of bariatric-metabolic surgery is approx. 0.1–0.3% [37]. In addition to acute complications of surgery (e.g., pulmonary embolisms, staple line leaks and fistula), metabolic surgery can also increase the risk of micronutrient deficiencies, skin wrinkling, weight regain, addiction, suicidality and suicides in the longer term.

Within 5 years, about 22% of patients present with weight regain after bariatric-metabolic surgery [38]. Therefore, close follow-up is essential for the long-term success of metabolic-bariatric surgery and the avoidance of complications. In particular, there is evidence, that long-term complication rates depend on patients’ follow-up adherence. In accordance with the guidelines, follow-up appointments should be made after 1, 3, 6, 12, 18 and 24 months and then annually.

## Surgical procedures

Current surgical procedures differ significantly in their complexity and weight loss outcomes. Individualized selection procedure takes into account initial weight, concomitant diseases, patient preferences and technical feasibility. The most common methods are sleeve gastrectomy (SG) (approx. 60% of procedures) and Roux-en-Y gastric bypass (RYGB). According to a recent retrospective cohort study, sleeve gastrectomy is associated with a more favorable risk profile than RYGB with regard to mortality, complications and reinterventions [39]. However, sleeve gastrectomy as primary intervention is inferior to RYGB in terms of weight loss (average weight loss at 1 year 25% vs. 31% for RYGB) and T2D remission rates in the short and long terms [40].

## Bariatric-metabolic surgery—sex-specific considerations

According to a recent systematic review and meta-analysis, bariatric procedures lead to higher absolute weight loss in men, who usually present with higher baseline BMI, and higher percentage of excess weight loss (%EWL) in women [41]. %EWL is calculated using an ideal body weight of BMI 25 kg/m^2^ as follows: (pre-operative body weight–follow-up body weight)/(pre-operative body weight–ideal body weight) × 100.

However, a matched-pair cohort analysis demonstrated that bariatric surgery results in comparable short- and mid-term efficacy in men and women, and is associated with similar rate and severity of postoperative complications between sexes. These findings suggest bariatric surgeons not to consider sex for patient selection in bariatric surgery [42]. However, short-term mortality as well as long-term mortality was higher in men compared to women according a systematic literature review [43].

In a comparison of SG and RYGB, patient sex and age significantly impacted weight loss in a procedure-dependent manner and should be considered when choosing between RYGB and SG [44]. Men under 40 and women over 50 years achieved less weight loss following SG compared to RYGB, whereas men over 40 years and women under 50 years experienced similar weight loss with either procedure [44].

It is important to note that bariatric surgery has shown to improve sex hormone profiles, ovulation and female sexual dysfunction [45]. This is of particular relevance in women < 40 years of age. However, it is recommended that pregnancy should be avoided for approximately 12–24 months following bariatric surgery [37], since rapid weight loss is associated with higher rates of nutritional deficiencies and obstetrics complications including higher incidence of still birth [46]. Therefore, effective contraception is obligatory. Due to alterations within the GI system and changes in pharmacokinetics following surgery, the use of oral combined hormonal contraceptives is not advised in postbariatric patients [47].

## Discussion and conclusion

Obesity is the result of an imbalance between energy expenditure and caloric intake. Although the importance of genetic determinants for the variance of body mass index (BMI) is about 60–70%, overeating, lack of exercise, and psychosocial stress are modifiable risk factors. To reduce obesity-associated comorbidities, a permanent reduction in body weight of (at least) 5–10% is recommended. A significant reduction of cardiovascular endpoints can be achieved with a weight reduction of more than 10% of the initial weight. Therapeutic measures in the context of an escalating stepwise approach should include strategies for targeted weight reduction and long-term weight maintenance.

It is interesting to note gender differences in response to both dietary/lifestyle and pharmacological interventions. The greater response in females to pharmacological interventions may be explained by physiological variations in pharmacokinetics and by slightly higher dose exposures in relation to body weight which consecutively results in a greater drug response. However, it is worth noting that even at similar exposures of the GLP-1 receptor agonist liraglutide, women lost more weight than men. These findings are consistent with data about semaglutide-associated gastrointestinal adverse events (AEs). Despite being dose dependent in general, AEs were reported to be more frequent in females at similar levels of exposure. However, so far, these differences have not been reported consistently enough to conduct sex-specific dosing recommendations and only few studies assessed the effect of sex on the mode of action of anti-obesity drugs. It is less obvious why men respond better to lifestyle interventions. It might be related to social factors and sex differences in the decision to participate in clinical research trials.

Data on the impact of sex on the effectiveness of bariatric-metabolic surgery are scarce and even more conflicting. For clarification, sex-stratified results and changes in body composition should always be specified and reported separately in future studies.
